# Editorial: A contemporary look at allergic rhinitis treatments: where are we heading?

**DOI:** 10.3389/falgy.2024.1459032

**Published:** 2024-07-18

**Authors:** Davor Plavec, Claudio Andaloro, Giorgio Ciprandi, Ignazio La Mantia, Cesare Miani, Attilio Varricchio

**Affiliations:** ^1^ Faculty of Medicine, Josip Juraj Strossmayer University of Osijek, Osijek, Croatia; ^2^ Department of Medical and Surgical Sciences and Advanced Technologies “GF Ingrassia” ENT Section, University of Catania, Catania, Italy; ^3^ Allergy Center, Casa di Cura Villa Montallegro, Genoa, Italy; ^4^ Department of Otolaryngology, University Hospital of Udine, Udine, Italy; ^5^ University of Molise, Campobasso, Italy

**Keywords:** ARIA (allergic rhinitis and its impact on asthma), allergic rhinitis (AR), multimorbidity (MM), treatment, management—healthcare

**Editorial on the Research Topic** A contemporary look at allergic rhinitis treatments: where are we heading?

Allergic rhinitis (AR) is a chronic inflammatory condition of the nasal mucosa triggered by allergens such as pollen, dust mites, and pet dander. It significantly affects the quality of life of millions globally, manifesting in symptoms like nasal congestion, itching, sneezing, and rhinorrhea. In the realm of allergy and immunology, the intricate relationship between allergic rhinitis (AR) and asthma has been a focal point of research, emphasizing the need for integrated management strategies. Recent advancements in the understanding of AR and its association with other allergic conditions, such as asthma, have opened new avenues for treatment (1–4). This editorial delves into contemporary research on AR treatments, highlighting key findings from recent studies, including the ARIA-MEDALL hypothesis by Bousquet et al., and discussing their implications for future management strategies (1).

The study by Bousquet et al. (1) presents a pivotal examination of the distinct characteristics of rhinitis associated with asthma vs. rhinitis alone, advancing the ARIA-MEDALL hypothesis. This research underscores the importance of differentiating these conditions in clinical practice to tailor more effective treatment strategies. The ARIA guidelines have evolved significantly, advocating for a comprehensive approach to allergic multimorbidities rather than treating these conditions as isolated entities. This perspective is crucial as it aligns with the growing understanding that allergic diseases often coexist, influencing each other's pathophysiology and treatment outcomes.

One of the studies within this Topic investigates the potential causal link between aspirin use and the reduced risk of hay fever or allergic rhinitis through a Mendelian randomization approach (Li et al.). By leveraging genome-wide association studies (GWAS) data, the researchers provide evidence suggesting that aspirin may play a preventive role in allergic rhinitis. This finding opens new avenues for considering aspirin not just as an anti-inflammatory agent but also as a potential preventive treatment for allergic conditions, warranting further exploration in clinical settings.

Exploring treatment efficacy, a comparative study on azelastine hydrochloride doses in perennial allergic rhinitis (PAR) patients revealed nuanced insights (Bousquet et al.). While both doses were well tolerated, the higher dose exhibited a statistically significant improvement over placebo, emphasizing the need for precise dosing to achieve optimal therapeutic outcomes. This research contributes to the ongoing discourse on balancing efficacy and safety in chronic allergy management.

Additionally, the integration of patient mHealth data and traditional epidemiological approaches has enriched our understanding of the phenotypic diversity in rhinitis and asthma. This combined methodology confirms the existence of varied phenotypes, necessitating personalized treatment approaches. Studies focusing on the multi-morbidity nature of allergic diseases, including the association of AR with conditions like conjunctivitis and eosinophilic esophagitis, further highlight the complexity and interconnectedness of allergic responses. With this problem deals another study from our Topic that concludes that the precise indications should be developed to arrive at a correct diagnosis of a specific phenotype and endotype of chronic rhinosinusitis (Canevari et al.). The various diagnostic pathways are needed to arrive at a correct therapeutic framing.

The next contribution to our Topic discuses the role of tolerogenic dendritic cells (tolDCs) in achieving immune tolerance in allergic rhinitis and reducing symptoms with the possibilities to use this pathway in developing different treatment strategies in allergic rhinitis (Kang et al.). It was shown that the activation of the TLR4/IRAK4/NF-*κ*B signaling pathway contributes to the release of inflammatory cytokines, and inhibitors of this signaling pathway induce the production of tolDCs to alleviate allergic inflammatory responses.

These contributions collectively advance our understanding of allergic rhinitis and asthma, highlighting the necessity of a holistic, patient-centered approach in managing these conditions. The research underscores the significance of differentiating phenotypes, exploring novel preventive strategies, and optimizing treatment protocols. As we continue to delve into the molecular and clinical aspects of these diseases, such integrated research efforts will be instrumental in shaping future guidelines and improving patient outcomes.

In summary, the studies featured in this Research Topic underscore the evolving landscape of allergic rhinitis and asthma research. By bridging clinical insights with genetic and epidemiological data, these contributions pave the way for more nuanced and effective management strategies. As we look forward to future research, the integration of diverse methodologies and personalized approaches will be key in addressing the complexities of allergic diseases and enhancing patient care.

This editorial brings to light the collaborative efforts and innovative research that drive advancements in the understanding and management of allergic rhinitis and asthma. Each study within this Topic not only contributes valuable findings but also sets the stage for future explorations in allergy research.
